# Inflammatory cytokines in pulmonary hypertension

**DOI:** 10.1186/1465-9921-15-47

**Published:** 2014-04-16

**Authors:** Alexandra Groth, Bart Vrugt, Matthias Brock, Rudolf Speich, Silvia Ulrich, Lars C Huber

**Affiliations:** 1 Division of Pulmonology, University Hospital Zurich, Rämistrasse 100, CH-8091 Zurich, Switzerland; 2 Institute of Surgical Pathology, University Hospital Zurich, Rämistrasse 100, CH-8091 Zurich, Switzerland

**Keywords:** Pulmonary hypertension, Inflammation, Immune cells, Experimental models, Cytokines, microRNAs

## Abstract

Pulmonary hypertension is an “umbrella term” used for a spectrum of entities resulting in an elevation of the pulmonary arterial pressure. Clinical symptoms include dyspnea and fatigue which in the absence of adequate therapeutic intervention may lead to progressive right heart failure and death. The pathogenesis of pulmonary hypertension is characterized by three major processes including vasoconstriction, vascular remodeling and microthrombotic events. In addition accumulating evidence point to a cytokine driven inflammatory process as a major contributor to the development of pulmonary hypertension.

This review summarizes the latest clinical and experimental developments in inflammation associated with pulmonary hypertension with special focus on Interleukin-6, and its role in vascular remodeling in pulmonary hypertension.

## Introduction

*Pulmonary hypertension* summarizes various conditions in which the blood pressure in the pulmonary circulation is significantly elevated. By definition, pulmonary hypertension is diagnosed when the mean pulmonary arterial pressure (mPAP) exceeds 25 mmHg as measured by right-heart catheterization. Since the first international conference by the World Health Organization (WHO) in Geneva in 1973, the classification of pulmonary hypertension was subjected to many changes. The current classification is based on the WHO-Conference in Nice (2013) [1] and separates the term pulmonary arterial hypertension (PAH) from pulmonary hypertension (PH) due to left heart disease, pulmonary disease, chronic thromboembolic pulmonary hypertension (CTEPH) and PH of miscellaneous etiologies. The current classification is summarized in the list ‘Updated clinical classification of pulmonary hypertension (Nice, 2013)’ below [1].

### Updated clinical classification of pulmonary hypertension (Nice, 2013) [1]

1. Pulmonary arterial hypertension (PAH)

1.1. Idiopathic PAH

1.2. Heritable PAH

1.2.1. BMPR2

1.2.2. ALK1, ENG, SMAD9, CAV1, KCNK3

1.2.3. Unknown

1.3. Drug- and toxin-induced

1.4. Associated with

1.4.1. Connective tissue diseases

1.4.2. HIV infection

1.4.3. Portal hypertension

1.4.4. Congenital heart diseases

1.4.5. Schistosomiasis

1’ Pulmonary veno-occlusive disease and/or pulmonary capillary hemangiomatosis

1” Persistent pulmonary hypertension of the newborn (PPHN)

2. Pulmonary hypertension due to left heart disease

2.1. Left ventricular systolic dysfunction

2.2. Left ventricular diastolic dysfunction

2.3. Valvular disease

2.4. Congenital/acquired left heart inflow/outflow tract obstruction and congenital cardiomyopathies

3. Pulmonary hypertension owing to lung diseases and/or hypoxia

3.1. Chronic obstructive pulmonary disease

3.2. Interstitial lung disease

3.3. Other pulmonary diseases with mixed restrictive and obstructive pattern

3.4. Sleep-disordered breathing

3.5. Alveolar hypoventilation disorders

3.6. Chronic exposure to high altitude

3.7. Developmental abnormalities

4. Chronic thromboembolic pulmonary hypertension (CTEPH)

5. Pulmonary hypertension with unclear multifactorial mechanisms

5.1. Hematologic disorders: chronic haemolytic anemia, myeloproliferative disorders, splenectomy

5.2. Systemic disorders: sarcoidosis, pulmonary histiocytosis, lymphangioleiomyomatosis

5.3. Metabolic disorders: glycogen storage disease, Gaucher disease, thyroid disorders

5.4. Others: tumoral obstruction, fibrosing mediastinitis, chronic renal failure, segmental PH

The pathophysiological mechanisms of pulmonary hypertension are not fully understood. Despite the clinical heterogeneity of the entities listed in ‘Updated clinical classification of pulmonary hypertension (Nice, 2013)’ [1] a common pathway resulting from a combination of genetic susceptibility and environmental factors seems to play a pivotal role in the pathogenesis of pulmonary hypertension. This pathway is characterized by vasoconstriction due to constrictive agents such as endothelin-1 [2], an imbalance of vasodilators (e.g. nitric oxide (NO) and prostacyclin) (e.g. endothelin-1) microthrombosis as well as vascular remodeling. Depending on the specific entity that causes the elevation of pulmonary pressure, these three factors are present in most forms of pulmonary hypertension. Oral anticoagulation and specific vasodilators are employed to address vasoconstriction and in situ thrombosis. However, in pulmonary hypertension the currently available drugs are insufficient to reverse vascular remodeling. Vascular remodeling is characterized by smooth muscle cell proliferation, hypertrophy of the medial layer, arteriolar muscularization and endothelial cell proliferation. Numerous factors have been identified that might trigger ongoing remodeling of the vessel wall but the bone morphogenetic protein receptor type II (BMPR2), which is predominantly expressed on pulmonary endothelium and smooth muscle cells, is considered to be the master regulator of vascular remodeling in pulmonary hypertension. Mutations or non-genetic alterations, such as the downregulation of this receptor, might lead to the vasculopathic lesions observed in patients with pulmonary hypertension. In up to 70% of familial PAH and in up to 30% of idiopathic PAH patients are carriers of BMPR2 mutations.

## Review

Evidence from animal models and studies in patients with pulmonary hypertension suggest that inflammation contributes to the development of pulmonary hypertension, in particular in PAH. In lung biopsies from patients with PAH, mononuclear cells are often observed in plexiform lesions, mainly consisting of T cells, macrophages and, to a lesser extent B cells [3]. A recent study revealed that the degree of perivascular inflammation correlates with both vascular wall thickness as well as mPAP [4]. The increased prevalence of PAH in patients with inflammatory diseases like thyroiditis [5] and in autoimmune disorders including connective-tissue diseases [6] further indicates an important role for the inflammatory process in the pathogenesis of the disease.

### Monocytes & macrophages

Increased numbers of macrophages are present in pulmonary lesions from patients with severe PAH [7]. Activation of macrophages induces the release of IL-1β, IL-6, tumor necrosis factor-α (TNF-alpha), and IL-10, which all play an important role in the pathogenesis of PAH [8]. Furthermore activated macrophages may present antigens to T cells resulting in T-cell activation and T-cell derived cytokine production, which further facilitates the inflammatory process associated with PAH [9]. Macrophages in mice with hypoxia-induced PH seem to switch their phenotype in a more activated type due to hypoxia and upregulate expression of genes involved in inflammatory processes (i.e. IL-1β, IL-13) [10]. Interestingly this switch may be caused by IL-6, one of the major elevated cytokine in PAH [11].

### T cells

T cells are increased in pulmonary vasculature in lungs from PAH patients. Cytotoxic CD8+ T cells even constitute the major part of the inflammatory component in plexiform vascular lesions. The nuclear factor of activated T cells (NFAT), a transcription factor that promotes cytokine gene transcription, is upregulated in PAH, leading to increased levels of cytokines, a main feature of PAH [12]. T cell deficient rats are more likely to develop PAH and deficiency of CD8+ T cells in PAH patients correlated with a worse survival, which indicate that T cells play a protective role during the development of PH [13]. Various pathways are likely to generate this protective effect, for example Treg (T regulatory) cells might prevent the development of pulmonary hypertension and margin endothelial injuries, through the upregulation of BMPR2 in lung tissue [14]. T cells have been shown to downregulate the macrophage-mediated inflammatory angiogenesis in the lung [7].

### B-cells

B-cell differentiation is stimulated by CD4+ T helper (Th) cells. These stimulated B cells produce autoantibodies which may explain the increased levels of antinuclear antibodies generally found in PAH patients [15]. Compared with non idiopathic PAH patients, B cells in peripheral blood from idiopathic PAH patients show a different RNA expression profile suggesting that in PAH patients B cells are activated [16].

**Figure 1:**
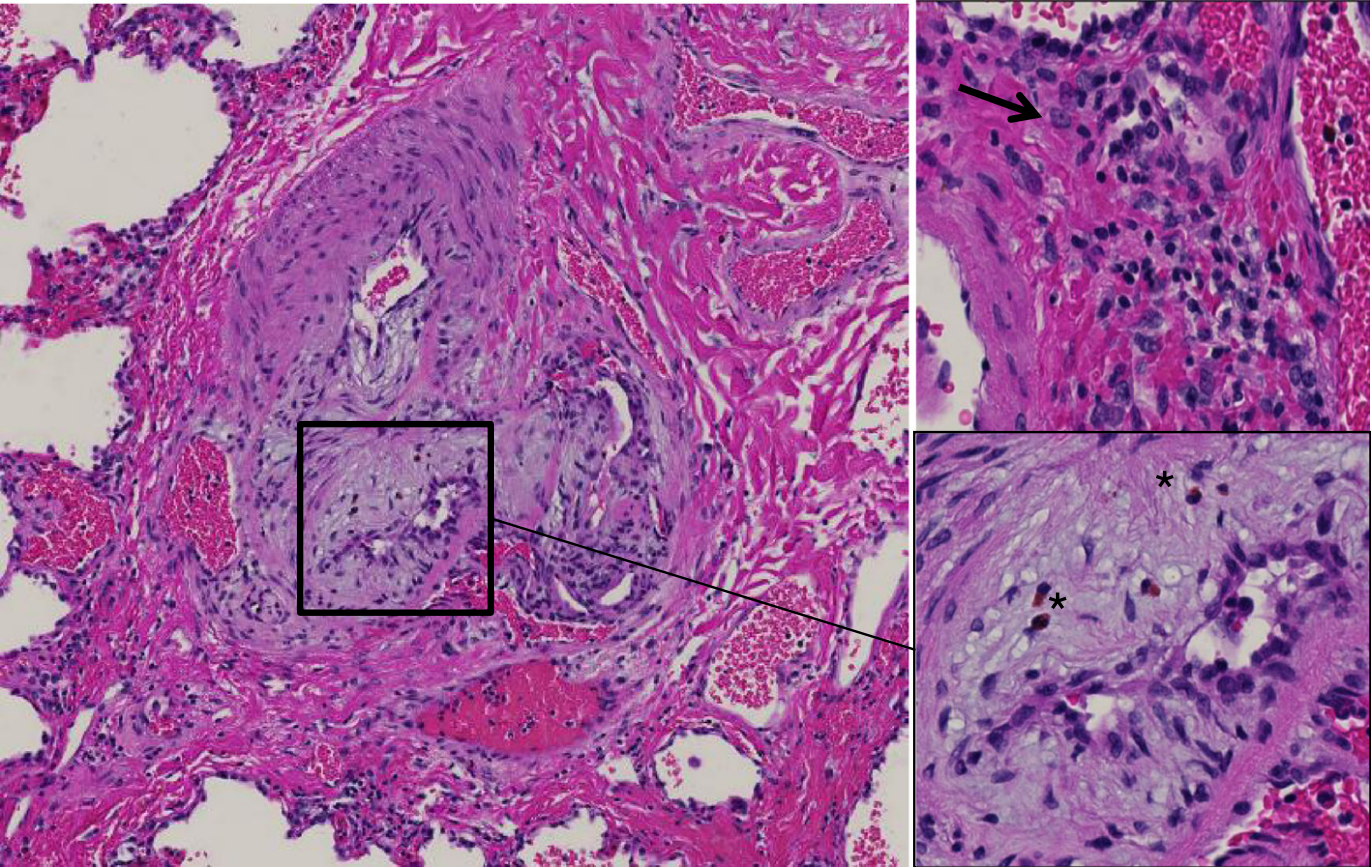
**Plexiform lesion in a patient with PAH.** Complex vascular lesion with perivascular fibrosis and infiltration by lymphocytes, plasma cells (arrow head) and eosinophils (*). HE staining.

Figure 1 shows the different inflammatory cells present in vasculopathic lesions of a patient with PAH.

### Cytokines

Cytokines represent a large group of signaling proteins that are produced and secreted by cells of the immune system and regulate numerous biological processes including inflammation, immunity and hematopoiesis. Cytokines are specific mediators that interact in an autocrine, paracrine or endocrine fashion.

Cytokines emerged as major contributing factors in the pathogenesis of pulmonary hypertension [17-19]. In addition, cytokines might act as biomarkers both for diagnosis and clinical outcome of patients with pulmonary hypertension. Here we review experimental results and clinical data of the most important cytokines in pulmonary hypertension. Several novel experimental and transgenic models have been described in the context of pulmonary hypertension [20] but it is unclear whether the findings in these models can be extrapolated to the human situation. The two best established models to date are the monocrotaline (MCT) and hypoxia induced model. Increased vascular remodeling has been observed by addition of an angiogenesis inhibitor, a modifying extension known as the “Sugen hypoxia” model. This model is promising to become a more physiological surrogate of the human disease. However, also in this model little is known about the contribution of inflammation.

### Specific cytokines

#### IL-1β

*Clinical data:* Elevated serum levels of IL-1beta were found in PAH patients and correlate with a worse outcome [21]. In a case report, the IL-1beta receptor antagonist Anakinra was shown to resolve pulmonary hypertension in a patient with Adult-Onset Still’s Disease [22].

*Experimental evidence*: In hypoxia-induced pulmonary hypertension and in the MCT model, data on IL-1β were found to diverge: in the MCT model, high levels of IL-1β were measured and, conversely, treatment with an IL-1β receptor antagonist reduced pulmonary hypertension and right ventricular hypertrophy, while no such findings were reported in the hypoxia mouse model [23]. This difference might be due to the action of MCT, a pyrrolizidine alkaloid with highly toxic and, potentially, inflammatory effects. In some studies, a link between levels of IL-1β and prostacyclins, in particular PGI2, was described: PGI2 is a metabolite of arachidonic acid with vasodilating and antiproliferative properties. The vasodilating effects are mediated through the second messenger cyclic adenosine monophosphate (cAMP). Patients with pulmonary hypertension have significantly decreased expression of endogenous PGI2 [24]. Interestingly, IL-1β enhances the expression of PGI2 in human pulmonary artery smooth muscle cells [25]. Similarly, in rat PASMC, IL-1-β increased the expression of PGI2 and 6-keto-PGF1α, a stable metabolite of PGI2 [26]. The increased expression of PGI2 might represent an endogenous response to the inflammatory injuries in the lung tissue. Itoh et al. measured an increase of the cyclooxygenase (COX)-2 mRNA in PASMC treated with IL-1β [26]. COX-2 is a key enzyme in the regulation of prostaglandin synthesis. Bradbury et al. showed that IL-1β induces COX-2 [27] an, in a follow-up paper, the authors showed that adenylyl cyclase, which converts adenosine triphosphate (ATP) to cAMP is downregulated by IL-1β. Moreover, accumulation of cAMP was attenuated in response to PGI2 analogues in human PASMCs, which is presumably due to COX-2 induction [28].

IL-18, a pro-inflammatory cytokine and member of the IL-1 family, is activated by the cleavage of IL-1β–converting enzyme, generating the biologically active IL-18. IL-18 is elevated in the patients with PAH and there is evidence that abnormal levels of IL-18 play a role in vasculopathy of the pulmonary circulation [29]. A recent study demonstrated that vascular injury may lead to an upregulation of IL-18 from PASMC of the medial vessel layer. IL-18 acts through an autocrine or paracrine effect on smooth muscle cells via its receptor, IL-18Rα, causing proliferation and recruitment of other smooth muscle cells. These mechanisms contribute to transmigration of PASMC and to hypertrophy of the medial vessel layer [29].

A recent study demonstrated that vascular injury may lead to an upregulation of IL-18 from PASMC of the medial vessel layer. IL-18 acts through an autocrine or paracrine effect on smooth muscle cells via its receptor, IL-18Rα, causing proliferation and recruitment of other smooth muscle cells. These mechanisms contribute to transmigration of PASMC and to hypertrophy of the medial vessel layer [29].

*Potential implications*: These data implicate that IL-1β appears to have deleterious effects for the development and progression of pulmonary hypertension. The exact mechanisms, however, remain unclear and therapeutic inhibition of IL-1β is limited to anecdotal case reports precluding therapeutic use at this moment.

#### IL-6

IL-6 is an important mediator in hepatic acute phase response [30] and is produced by inflammatory cells, i.e. monocytes and T-lymphocytes. As suggested by recent publications, IL-6 might be one of the most important cytokines involved in the pathogenesis of PAH and hypoxia-induced pulmonary hypertension.

*Clinical data:* Serum levels are significantly higher in patients as compared with normal controls [31]; the levels were found to correlate with patients survival and levels of IL-6 also turned out to be a better predictor for survival than traditional clinical tests (e.g. the 6-minute walking distance and hemodynamic measurements) [21,32]. Moreover, IL-6 seems to have a strong impact on the development of pulmonary hypertension in COPD. COPD patients with pulmonary hypertension had higher plasma levels than those without pulmonary hypertension and the levels of IL-6 correlated with the mPAP [33]. A further association was found between the presence of pulmonary hypertension in COPD patients and polymorphisms of the IL-6 gene: patients with the GG phenotype (-174G/C) of the IL-6 gene had higher pulmonary pressure than patients with the CC or GC phenotype [33,34]. These data indicate that variations in the genes encoding inflammatory cytokines might contribute to the development of pulmonary hypertension. About 6% of patients with liver cirrhosis develop PAH (portopulmonary hypertension, PPHTN) [35]. In these patients, IL-6 was found to be significantly increased compared to cirrhosis patients without elevation of the pulmonary pressure [36].

*Experimental evidence:* Increased levels of IL-6 mRNA were measured in MCT rats that developed pulmonary hypertension and right ventricular hypertrophy (RVH). When these rats were treated with immunosuppressive steroids decreased levels of IL-6 and reduced pulmonary pressures and RVH were measured [37]. Similar findings were obtained in mice by injections of supraphysiological doses of IL-6 that resulted in pulmonary hypertension, an effect that was even pronounced under hypoxic conditions [38]. The most convincing data for the role of IL-6 were reported by Steiner et al. that employed transgenic mice overexpressing IL-6. These animals showed enhanced muscularization both of the proximal arterial tree and in the distal arteriolar vessels and were found to have occlusive neointimal angioproliferative lesions, mostly consisting of endothelial cells and T-lymphocytes. These vasculopathic changes corresponded to the increase of right ventricular systolic pressure and RVH [39,40].

As mentioned before, BMPR2 mutations might be found in about 70% of familial PAH and in up to 30% of idiopathic PAH patients. Of interest, however, dysregulation of the BMPR2 receptor has also been found in other forms of pulmonary hypertension. In an experimental model, Takahashi et al. found a significant downregulation of BMPR2 in rodents exposed to hypoxia [41]. Since these changes could not be correlated with adequate changes of the corresponding mRNA levels, a finding also confirmed by the MCT model of experimental pulmonary hypertension [42], Brock et al. identified a posttranscriptional mechanism to be responsible for the downregulation of BMPR2, involving IL-6, the signal transducer and activator of transcription STAT3 and the microRNA cluster 17/92 [43].

Subsequent studies showed that specific inhibition of these microRNAs by antagomiRs were found to restore functional levels of BMPR2 and to inhibit or even reverse the vascular remodeling and subsequent hemodynamic alterations [44,45].

In addition, IL-6 might contribute to vascular remodeling also through other, miR-independent pathways. For example, it was shown that elevated levels of IL-6 resulted in an upregulation of vascular endothelial growth factor receptor II (VEGFR2) and matrix metalloproteinase-9 (MMP-9), an endopeptidase that promotes angiogenesis through regulation of cell attachment, proliferation, and migration. MMP-9 itself was found to upregulate VEGFR2, whereas levels of the ligand, VEGF, are increased by IL-6 directly. As such, high levels of IL-6 continuously activate the proliferation of PASMCs and probably trigger the transformation of pulmonary endothelial cells to pulmonary arterial smooth muscle cells [39].

**Figure 2:**
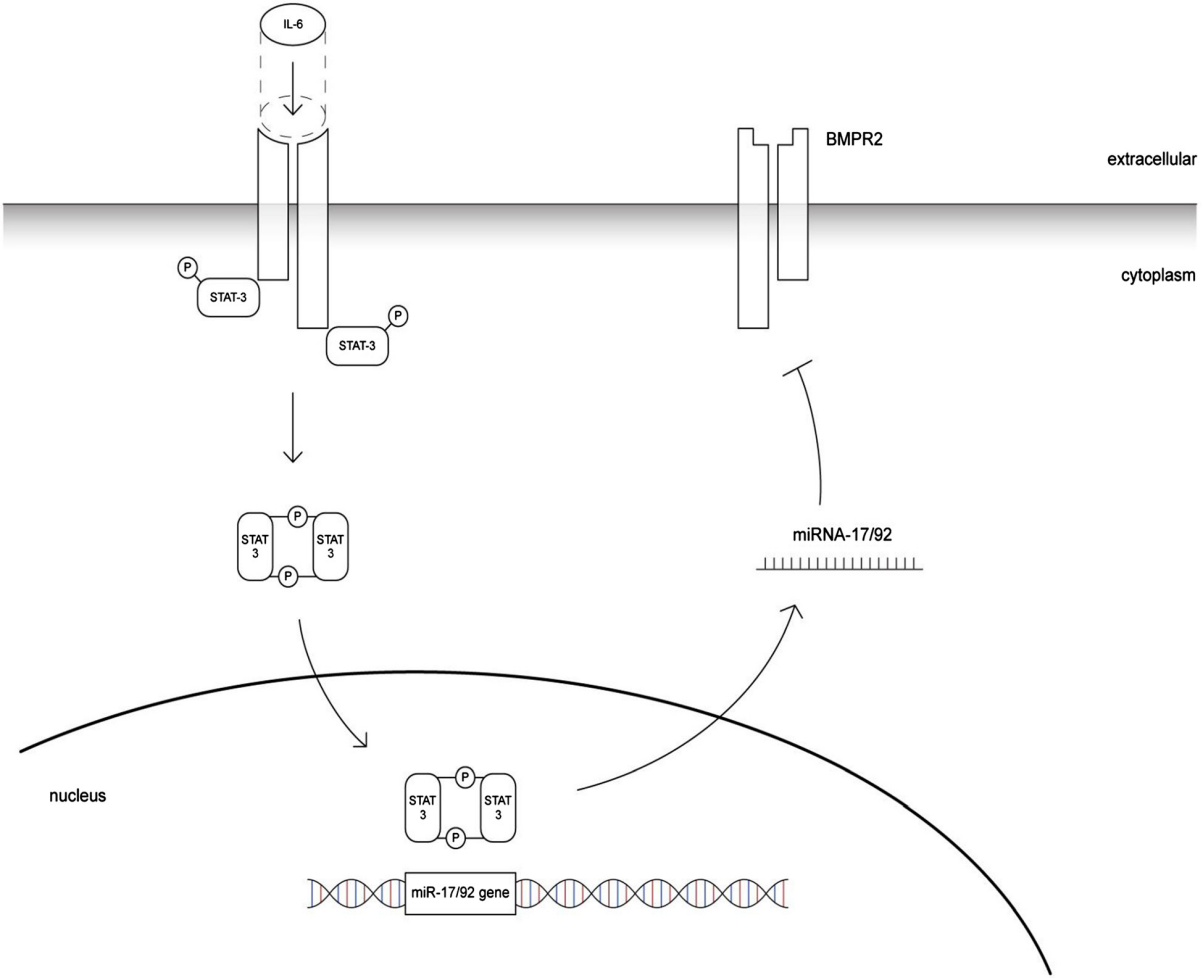
**Proposed mechanism of BMPR2 downregulation by IL-6.** The binding of IL-6 to its receptor triggers the phosphorylation of STAT3. Phosphorylated STAT3 forms a dimer and translocates into the nucleus, where it activates the transcription of the miR-17/92 cluster. The mature miRNA transfers into the cytoplasma and, by binding to the target mRNA, silences BMPR2.

*Potential implications*: IL-6 seems to be one of the most important inflammatory cytokines in the development of PAH, and in particular of hypoxia-induced pulmonary hypertension. The IL6 - STAT3 - miR-17/92 - BMPR2 pathway is an attractive tool that contributed to the understanding of the pathogenesis of the pulmonary arterial remodeling and, in the future, might be further translated into the development of a causative treatment (Figure 2).

#### IL-8

*Clinical data:* Elevated serum levels of IL-8 were found in PAH patients and were also described as predictor of survival in PAH patients [21]. IL-8 is thought to play an important role in the development of PAH, especially in early phases of vascular remodeling. IL-8 is known to have proangiogenic and antiapoptotic activities and acts as a growth factor for endothelial cells [46]. These effects could also explain why patients with PAH in association with connective tissue diseases show higher IL-8 serum levels than patients without PAH [47]. Levels of IL-8 are elevated in early stages of high altitude pulmonary edema implicating that IL-8 might be involved in the hypoxic pressure response of pulmonary vessels [48].

*Experimental evidence:* Downregulation of the C-C chemokine receptor type 7 (CCR7), a regulator of lymphocyte trafficking [49], was described in patients with PAH. This deficiency leads to perivascular infiltration of T and B cells in mouse lungs, similarly to the findings observed in human PAH [50]. CCR7 -/-mice show elevated mRNA levels of IL-12 that - since IL-12 acts upstream of IL-8 in bronchial epithelial cells - [51] triggers the release of IL-8 [52].

#### IL-10

IL-10, released by T-cells, is one of the most important anti-inflammatory cytokines that inhibit overreaching inflammatory processes.

*Clinical data:* Elevated levels of IL-10 are found in patients with PAH, which could serve as counterregulating mechanisms against the inflammation in lung tissue. The elevated IL-10 levels were found to be inversely correlated with prostacyclin agonists therapy, i.e. patients under a PH-target therapy with prostacyclin agonists showed higher levels of IL-10 compared to patients without such therapy [21]. Conversely, PAH patients showed a significant decrease in IL-10 expression following cardiopulmonary bypass operation [53].

*Experimental evidence:* Ito et al. demonstrated that injections of IL-10 reduced the mean pulmonary arterial pressure in MCT rats and significantly improved survival [54].

*Potential conclusion:* Whether these observations reflect “true mechanisms” or represent an abnormal response remain unclear at the moment. In fact, since intravenous prostacyclin agonists are indicated for severe disease the correlation between levels of IL-10 and intravenous PH target therapy might be biased and it cannot be excluded that levels of IL-10 correlate with the severity of PAH [21,22]. Experimental data, however, suggest a protective role of the anti-inflammatory cytokine IL-10.

#### IL-13

*Experimental evidence:* According to previous research data, IL-13 acts as an important mediator of cell proliferation and tissue remodeling in lungs [55]. In experimental pulmonary hypertension the role of IL-13 remains ambiguous. IL-13 acts mainly through two receptors: the low affinity receptor IL-13Rα1 and the high affinity receptor IL-13Rα2. The IL-13Rα2 is a ‘decoy’ receptor and acts as a strong and selective IL-13 signaling inhibitor. Both IL-13 and IL-13Rα2 are found highly expressed in pulmonary vessels of PAH patients. Hecker et al. showed by in vitro experiments that addition of Il-13 decreased proliferation of PASMC, an effect that was pronounced by silencing IL-13Rα2. Conversely, Graham et al. demonstrated that infection with the parasite Schistosoma mansoni resulted in PAH and remodeling of pulmonary arteries. Since this finding was pronounced in mice lacking IL-13Rα2, the pro-proliferative effects on pulmonary vessels observed in Schistosomiasis are probably mediated by the eosinophilic effector cytokine IL-13 [56].

Further evidence for a role of IL-13 to promote vascular remodeling in pulmonary hypertension comes from Cho et al. that investigated an IL-13 – IL-13Rα2 – Arginase 2 (Arg 2) pathway. Arg2 is a key enzyme of the L-arginine metabolism and was found to be induced by IL-13 in lung tissue from mice [57]. It is thought that Arg2 contributes to pulmonary hypertension mainly by competing with nitric oxide (NO)-synthase for the substrate arginine, leading to reduced bioavailability of the vasodilating NO [58]. Moreover, the enzymatic reaction of Arg2 itself appears to generate pro-proliferative factors [59]. Consistent with these findings, in Arg2 -/-mice overexpressing IL-13 remodeling of pulmonary arteries was found to be decreased [60].

*Potential conclusion:* IL-13 promotes the development of pulmonary hypertension via an IL-13 - IL-13Rα2 - Arg2 pathway leading to an imbalance of NO homeostasis and increased muscularization of pulmonary arteries. However, the experimental data show both protective and deleterious effects of IL-13 and it is too early to make conclusions on the potential use of IL-13 and its pathways as therapeutic target for pulmonary hypertension.

#### TNF-α

*Clinical data:* Similarly to other inflammatory cytokines, elevated serum levels of tumor necrosis factor (TNF)-α were described in PAH patients [21]. Moreover, COPD patients with pulmonary hypertension show significantly higher TNF-α and C-reactive protein levels than COPD patients without pulmonary hypertension, further corroborating the role of COPD as an inflammatory systemic disease [61].

*Experimental evidence:* When used in high concentrations, TNF-α suppresses the mRNA expression of the vasodilating PGI2 [26]. Injections of TNF-α to rats also increased vascular reactivity, which might contribute to pulmonary hypertension [62]. Similarly, TNF-α over expression in alveolar type II cells resulted in chronic pulmonary inflammation, septal destruction, bronchiolitis and pulmonary hypertension [63].

Sutendra et al. hypothesized that increased levels of TNF-α may lead to a decrease of pyruvate dehydrogenase (PDH). PDH is a mitochondrial gate-keeping enzyme and may play an important role by making pulmonary arterial smooth muscle cells resistant to apoptosis. It could be demonstrated that the PDH activity was significantly decreased in cells treated with TNF-α, while MCT-treated rats that were injected with Etanercept (a TNF-α antagonist) were found to be protected from development of PAH [64]. In another study, rats treated with a TNF-α blocker (rhTNFRFc) showed some amelioration in pulmonary hemodynamics, right ventricular hypertrophy and pulmonary inflammation [65] and in pigs with endotoxemic-shock-induced pulmonary hypertension, Etanercept was able to lower both pulmonary arterial pressure and pulmonary vascular resistance compared to pigs without Etanercept therapy [66]. Other studies using TNF-α - antagonists, however, could not confirm an improvement of pulmonary hypertension [67,68].

*Potential conclusion:* TNF-α might play an important role in the development of pulmonary hypertension, even though the concrete mechanisms remain unknown. Interestingly some studies show that TNF-α blockers ameliorate pulmonary pressure, while other studies found no significant effects.

## Conclusions

Inflammatory cytokines seem to play a crucial role in the development of pulmonary hypertension. However, while experimental research has contributed a lot to our understanding of the pathogenesis and development of this devastating disease, it remains difficult to provide an integrative pathway for the different identified factors and to translate these findings to human pulmonary hypertension, which remains a challenge for future research in the field. Since the cytokines discussed in this article and the cells that release them and respond to them probably form a complex network with different signaling pathways involved, many conclusions on the role of inflammatory cytokines for pathogenesis and treatment of pulmonary hypertension remain speculative so far. Moreover, due to the multiple and redundant activation of pathways and the interaction of many cytokines, targeting one specific factor might not prove successful in a clinical setting.

It is the authors’ view that the best-investigated and most promising cytokine to date is IL-6, in particular for the development of hypoxia-induced pulmonary hypertension. Research focusing on the pathway of IL-6, involving the action of microRNAs and regulation of the expression of BMPR2 thus is ongoing to extend these findings to other forms of pulmonary hypertension or the use of these factors as surrogate markers for the disease.

## Abbreviations

Arg2: Arginase 2; ATP: Adenosintriphosphate; BMPR2: Bone morphogenetic protein receptor type 2; cAMP: Cyclic adenosine monophosphate; CCR7: C-C chemokine receptor type 7; COPD: Chronic obstructive pulmonary disease; COX: Cyclooxygenase; CTEPH: Chronic thromboembolic pulmonary hypertension; ET-1: Endothlin-1; IL: Interleukin; IL-13Rα1: Interleukin-13 receptor α1; IL-13Rα2: Interleukin-13 receptor α2; IL-18Rα: Interleukin-18 receptor α; 6-keto-PGF1α: 6-keto Prostaglandin F1α; MCI: 1-[o-(m-methoxyphenyl)ethyl]phenoxy]-3-(dimethylamino)-2-propyl hydrogen succinate hydrochloride; MCT: Monocrotaline; MMP-9: Matrix metallopeptidase 9; mPAP: Mean pulmonary arterial pressure; miR: Micro RNA; mRNA: Messenger RNA; NFAT: Nuclear factor of activated T-cells; NO: Nitric oxide; NYHA: New York Heart Association; PAH: Pulmonary arterial hypertension; PASMC: Pulmonary artery smooth muscle cells; PDH: Pyruvate dehydrogenase; PGI2: Prostaglandin I2; PPHTN: Portopulmonary hypertension; rhTNFRFc: Recombinant tumor necrosis factor receptor:Fc fusion protein; RVH: Right ventricular hypertrophy; STAT3: Signal transducer and activator of transcription 3; TNF-α: Tumor necrosis factor-alpha; TGFβ2: Transforming growth factor-beta 2; VEGF: Vascular endothelial growth factor; VEGFR2: Vascular endothelial growth factor receptor 2.

## Competing interests

The project “the role of microRNAs in pulmonary hypertension: diagnosis and treatment” is supported by the Swiss National Science Foundation (SNF 31003A_144212) and the Zurich Lung Foundation. The authors declare that they have no competing interests.

## Authors’ contributions

AG performed literature search and wrote drafts and revisions of the manuscript. BV provided Figure 2 and assisted to write the final version of the mansucript. MB reviewed all versions of the manuscript and assisted to write the final version. RS reviewed all versions of the manuscript and assisted to write the final version. SU reviewed all versions of the manuscript and assisted to write the final version. LH supervision of AG. assisted to write all drafts and revisions and wrote the final version. All authors read and approved the final manuscript.
